# Distinctive microRNA expression in early stage nasopharyngeal carcinoma patients

**DOI:** 10.1111/jcmm.12906

**Published:** 2016-08-04

**Authors:** Shuna Li, Lihua Hang, Yongming Ma, Chaoyang Wu

**Affiliations:** ^1^ Department of Otolaryngology and Head‐Neck SurgeryZhenjiang First People's HospitalThe Affiliated People's Hospital of Jiangsu UniversityZhenjiangJiangsuChina; ^2^ Department of AnesthesiaZhenjiang First People's HospitalThe Affiliated People's Hospital of Jiangsu UniversityZhenjiangJiangsuChina; ^3^ Department of Radiation OncologyZhenjiang First People's HospitalThe Affiliated People's Hospital of Jiangsu UniversityZhenjiangJiangsuChina

**Keywords:** microRNA, nasopharyngeal carcinomas, microarrays, early stage

## Abstract

The goal of this study was to investigate microRNAs (miRs) expression at different stages of nasopharyngeal carcinoma (NPC). MiR expression profiling at various stages of NPC was performed by miR array and further verified using quantitative real‐time RT‐PCR. Pathway enrichment analysis was carried out to identify the functional pathways regulated by the miRs. The expression of a selected group of identified miRs was verified in stage I NPC by *in situ* hybridization (ISH). A total of 449 miRs were identified with significantly different expressions between NPC tissues and normal pharyngeal tissues. Eighty‐four miRs were dysregulated only in stage I NPC, among which 45 miRs were up‐regulated and the other 39 were down‐regulated. Pathway enrichment assay revleaed that three significantly down‐regulated and three significantly up‐regulated miRs involved in 12 pathways associating with tumour formation and progression. Quantitative RT‐PCR confirmed the miR array result. In addition, the low expression levels of hsa‐miR‐4324, hsa‐miR‐203a and hsa‐miR‐199b‐5p were further validated in stage I NPC by ISH. This present study identifed the miR signature in stage I NPC, providing the basis for early detection and treatment of NPC.

## Introduction

Nasopharyngeal carcinoma (NPC) is a common type of cancer in Southeastern Asia and Africa. It is closely related to many viral, dietary and genetic factors 1, 2, 3. Intensity‐modulated radiation therapy and active anticancer agents are standard treatment options for NPC 4. In recent years, cancer stem cells and gene therapy are new concepts and promising strategies for NPCs, but these new technologies have yet to be applied in the clinic 5, 6. Similar to other types of malignant tumour, TNM stages of NPC are significantly correlated with the treatment efficacy and the prognosis of the disease. Stage I NPC is easier to treat while prognosis is very poor in late stage NPC 7, 8, 9. Therefore, it is important to understand the biomarkers and signatures of early stage NPC so that treatment can start right away.

MicroRNAs (or miRNAs) are short non‐coding RNAs involved in post‐transcriptional regulation of gene expression 10. They can be found in various organisms including animals, plants and viruses, and they play a key role in diverse biological processes, such as embryogenesis, differentiation and proliferation of cells, production of cytokines or apoptosis 10, 11. Since the initial observation, more and more miRNAs have been identified in mammalian cells and up to one‐third of all protein‐encoding genes are estimated to be regulated by these small molecules 12. Based on current literature, miRNA dysregulation plays a major role in head and neck/oral cancer 13. Identification of the dysregulated miRNAs in cancer (especially at early stages) offers great potential for early diagnosis and new therapeutic targets 14, 15. To that end, it is crucial to study the dysregulated miRNAs in NPC. Previous studies suggest the importance to study the relationship between miRNAs and NPC 16, 17, 18, 19, 20, 21. Also, some researches have been carried out to reveal with the relationship between miRNA expression and NPC radioresistance and recurrence 22, 23, 24, 25. To date, little has been known regarding the dysregulated miRNAs in early stage NPC.

In this study, we employed the Agilent Microarray platform to analyse miR expression in different stages of NPC. Interestingly, 84 miRs were found dysregulated only in stage I NPC. Pathway enrichment analysis and *in situ* hybridization (ISH) further revealed the cancerous pathways regulated by the identified miRs. We expect our results provide possible targets for the development of new gene therapies to treat NPC at early stages 26.

## Material and methods

### Tissue samples

All samples were obtained with approval of the Ethics Committee of the Affiliated People's Hospital of Jiangsu University. Nasopharyngeal carcinoma tissue samples were taken from poorly differentiated squamous NPC patients at different TNM stages before treatment at the Cancer Center of the Affiliated People's Hospital of Jiangsu University. Normal nasopharyngeal tissue samples were collected in the same hospital. Eight samples were obtained from eight NPC patients at different stages and two samples from normal nasopharyngeal tissues. Samples we used are listed in Table 1. Those 10 samples were further divided into five groups: Normal, stage I, II, III and IV for microarray analysis. According previous results, sample pooling does not significantly improve inferences. One can decrease the number of arrays required in an experiment without a loss of precision 27, 28. All tissues were fixed in 10% neutralized formalin and embedded in paraffin. Pathological types were confirmed by haematoxylin and eosin staining and immunohistochemically staining. TNM stages were judged according to the UICC/AJCC staging system for NPC, seventh edition (2009).

**Table 1:** The information of NPC samples

Sample	Gender	Age	TNM stage	Cancer stage
1	Male	68	T1N0M0	I
2	Male	56	T1N0M0	I
3	Male	49	T1N1M0	II
4	Female	59	T1N1M0	II
5	Male	73	T3N0M0	III
6	Female	67	T2N2M0	III
7	Male	73	T3N3M1	IV
8	Male	52	T2N3M0	IV

### RNA isolation and microRNA microarray hybridization

Total RNA was extracted and purified using RecoverAll^™^ Total Nucleic Acid Isolation Reagent (Ambion, Austin, TX, USA) following the manufacturer's instructions. RNA concentration and integration were examined by Agilent Bioanalyzer 2100 (Agilent Technologies, Santa Clara, CA, USA). The MiRs in total RNA were labelled using the miRNA Complete Labeling and Hyb Kit (Agilent Technologies) following the manufacturer's instructions. Each slide was hybridized with 100 ng Cy3‐labelled RNA using miRNA Complete Labeling and Hyb Kit (Agilent Technologies) in hybridization Oven (Agilent Technologies) at 55°C, 20 r.p.m. for 20 hrs according to the manufacturer's instructions. After hybridization, slides were washed in staining dishes (Thermo Shandon, Waltham, MA, USA) with Gene Expression Wash Buffer Kit (Agilent Technologies). Slides were scanned by the Agilent Microarray Scanner (Agilent Technologies) powered by the Feature Extraction software 10.7 (Agilent Technologies) with default settings. Raw data were normalized by Quantile algorithm, Gene Spring Software 11.0 (Agilent Technologies). After normalization, differentially expressed miRs were identified through Fold Change filtering.

### Real‐time quantitative PCR

To ascertain the microarray results, miR‐203a, miR‐199b‐5p, miR‐2117, miR‐4494, miR‐4502 and miR‐4324 were selected for quantitative real‐time RT‐PCR analysis. FAM‐labelled Taqman ABI probe‐based real‐time PCR assays for miR‐4324 (context sequence: CCCUGAGACCCUAACCUUAA), miR‐203a (context sequence: AGUGGUUCUUAACAGUUCAACAGUU), miR‐199b‐5p (context sequence: CCCAGUGUUUAGACUAUCUGUUC), miR‐2117(context sequence: UGUUCUCUUUGCCAAGGACAG), miR‐4494 (context sequence: CCAGACUGUGGCUGACCAGAGG) and miR‐4502(context sequence: GCUGAUGAUGAUGGUGCUGAAG) were carried out on: ABI 7900 HT Sequence Detection System according to the ABI Taqman microRNA assay protocol. U6 small nuclear RNA was used as the internal standard for determining the relative miRNA expression level. The reactions were incubated at 50°C for 2 min., 95°C for 10 min., followed by 40 cycles at 95°C for 15 sec., 60°C for 1 min. All PCR reactions were performed in triplicate. The 2^−ΔCt^ method was used as relative quantification measure of differential expression.

### MicroRNAs *in situ* hybridization

Locked nucleic acid (LNA) ISH on paraffin tissue sections was performed with a double 5′‐digoxigenin (DIG)‐labelled LNA probe specific for human miR‐4324, miR‐203a and miR‐199b‐5p (Exiqon, Woburn, MA, USA). 20 paraffin‐embedded sections came from 20 NPC patients (five in each NPC stage) were used for ISH analysis. First, paraffin‐embedded sections were deparaffinized in xylenes and then rehydrated through an ethanol dilution series. Slides were then treated with Proteinase K at 15 μg/ml for 10 min. at 37°C. Hybridization was performed at 54°C for the following: DIG labelled (U6) and double DIG (scrambled and miR‐4324, miR‐203a, miR‐199b‐5p), LNA‐modified oligonucleotide ISH probes. Positive probe labelling was blue/purple. Nuclei were visualized using Nuclear Fast Red counterstain (Vector Laboratories Inc., Burlingame, CA, USA).

## Results

### Distinctive microRNA expressions in NPC at different stages and nasopharyngitis tissues

**Figure 1:**
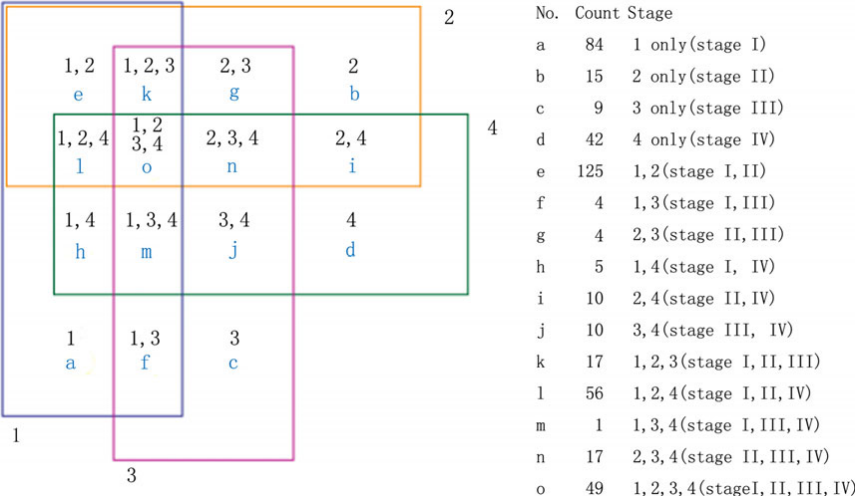
Venn diagram of differentially expressed miRNAs in stages of NPC. Nomber1, 2, 3, 4: stage I, II, III, IV and a‐o: miRNA number with diverse expression in different stage.

A total of 2006 human miRs were detected with the Agilent's microarray platform driven by Sanger miRBase (Release 19.0). The original data were analysed using Gene Spring Software (Agilent Technologies) after normalization for fold change to identify differentially expressed genes, based on the following selection criteria: fold change (linear) ≤0.5 or fold change (linear) ≥2. After the initial screening, 449 miRs were kept for the subsequent distinctive analysis. As shown in the Venn diagram (Fig. 1), some dysregulated miRs only appeared in certain stage, while others appeared in more than one stage of NPC.

### Distinctive microRNA expression in stage I NPC

We are interested in distinctive miR expression at various stages of NPC, especially in stage I NPC. Stage I is crucial for the formation of NPC and an important time‐point to intervene 11. In this study, we found total 84 miRs only dysregulated in stage I NPC (Fig. 1). Among those 84 miRs, 45 were up‐regulated and 39 were down‐regulated (Table 2). One miR can target hundreds of genes and one gene can be targeted by multiple miRs. To that end, we selected the most dysregulated miRs for the further analysis, according to highest or lowest FC values (*i.e*. high FC values obtained by up‐regulated miRs and lower FC values obtained by down‐regulated miRs). To evaluate the biological consequence of abnormal miR expressions, we employed the miRDB software 29, 30 to analyse targeted genes and functions. We also employed the Cytoscape software (The Cytoscape v1.1 Core runs on all major operating systems and is freely available for download from http://www.cytoscape.org/as an open source Java application.) to generate miRs function network. Using DAVID tools (The Database for Annotation, Visualization and Integrated Discovery v6.7) 31, 32 we acquired pathway enrichment from gene ontology. Through the KEGG pathway databases, we examined the pathway targets enrichment (*P* < 0.05) of down‐regulated miRNAs (hsa‐miR‐4324, hsa‐miR‐203a, hsa‐miR‐199b‐5p) (Table 3) and up‐regulated miRNAs (hsa‐miR‐2117, hsa‐miR‐4494, hsa‐miR‐4502) (Table 4).

**Table 2:** MicroRNAs only dysregulated in stage I NPC. 45 miRNAs were up‐regulated (left, FC ≥2) and 39 miRNAs were down‐regulated (right, FC ≤0.5)

Up‐regulated miRNAs	Fold change (NPC/Normal pharyngeal tissue	Down‐regulated miRNAs	Fold change (NPC/Normal pharyngeal tissue
hsa‐miR‐2117	35.19640078	hsa‐miR‐203a	0.00438528
hsa‐miR‐4502	28.42966709	hsa‐miR‐4324	0.013114843
hsa‐miR‐4494	24.85078121	hsa‐miR‐199b‐5p	0.015106744
hsa‐miR‐5686	6.234625357	hsa‐miR‐152	0.015655062
hsa‐miR‐3163	5.981554649	hsa‐miR‐532‐5p	0.021356314
hsa‐miR‐139‐5p	5.897478688	hsa‐miR‐214‐3p	0.02341181
hsa‐miR‐4436a	5.782638599	hsa‐miR‐132‐3p	0.02550163
hsa‐miR‐4674	5.673874972	hsa‐miR‐98‐5p	0.076613182
hsa‐miR‐4717‐3p	5.63948609	hsa‐miR‐199a‐5p	0.080598807
hsa‐miR‐4748	5.538848968	hsa‐miR‐10b‐5p	0.085482191
hsa‐miR‐518a‐5p	5.429137528	hsa‐miR‐148b‐3p	0.095244995
hsa‐miR‐3680‐3p	5.411772528	hsa‐miR‐6073	0.114204104
hsa‐miR‐30b‐3p	5.311856969	hsa‐miR‐199a‐3p	0.125620428
hsa‐miR‐4519	5.270859003	hsa‐miR‐193a‐3p	0.12792638
hsa‐miR‐1254	4.898350333	hsa‐miR‐151a‐3p	0.128389409
hsa‐miR‐4660	4.861456796	hsa‐miR‐487b	0.144085394
hsa‐miR‐4694‐3p	4.692768851	hsa‐miR‐128	0.145525102
hsa‐miR‐4707‐3p	4.658102545	hsa‐miR‐125a‐5p	0.1829238
hsa‐miR‐4697‐3p	4.632601224	hsa‐let‐7e‐5p	0.191501673
hsa‐miR‐4314	2.62042406	hsa‐miR‐99b‐5p	0.194760485
hsa‐miR‐339‐3p	2.605952703	hsa‐miR‐200b‐3p	0.277032588
hsa‐miR‐4526	2.599055786	hsa‐let‐7d‐5p	0.288889485
hsa‐miR‐1323	2.596089822	hsa‐miR‐22‐3p	0.325533856
hsa‐miR‐1469	2.504560567	hsa‐miR‐324‐5p	0.338455696
hsa‐miR‐6129	2.488412744	hsa‐miR‐365a‐3p	0.34912837
hsa‐miR‐3682‐3p	2.43621794	hsa‐miR‐374b‐5p	0.355823031
hsa‐miR‐1273c	2.435106719	hsa‐miR‐146b‐5p	0.370538563
hsa‐miR‐4673	2.377707584	hsa‐miR‐146a‐5p	0.371381254
hsa‐miR‐652‐5p	2.373619362	hsa‐miR‐664a‐3p	0.37268886
hsa‐miR‐4476	2.274244399	hsa‐miR‐23b‐3p	0.378322373
hsa‐miR‐424‐3p	2.249633838	hsa‐miR‐361‐5p	0.378351875
hsa‐miR‐4758‐5p	2.249353332	hsa‐let‐7f‐5p	0.404459317
hsa‐miR‐4257	2.203649232	hsa‐let‐7g‐5p	0.409047985
hsa‐miR‐4507	2.183758018	hsa‐miR‐425‐5p	0.409401672
hsa‐miR‐4470	2.169207555	hsa‐miR‐29a‐3p	0.420675499
hsa‐miR‐5088	2.168038536	hsa‐miR‐27b‐3p	0.440837238
hsa‐miR‐564	2.153669691	hsa‐miR‐3676‐3p	0.462586959
hsa‐miR‐4745‐5p	2.134746854	hsa‐miR‐1260b	0.46793252
hsa‐miR‐3605‐5p	2.12263866	hsa‐let‐7i‐5p	0.468495144
hsa‐miR‐3654	2.120827526		
hsa‐miR‐1273e	2.093393465		
hsa‐miR‐4481	2.076230617		
hsa‐miR‐550a‐3‐5p	2.033664087		
hsa‐miR‐4294	2.019948765		
hsa‐miR‐3945	2.013610577		

**Table 3:** Pathways enrichment and related genes of hsa‐miR‐4324, hsa‐miR‐203a and hsa‐miR‐199b‐5p (three down‐regulated miRs in stage I NPC)

KEGG_PATHWAY	Count	%	*P*‐value	Genes
Pathways in cancer	40	2.8531	0.001283	KITLG, GLI3, TPM3, TGFB2, LAMB4, PTK2, PAX8, PIK3CA, NKX3‐1, TPR, COL4A4, PRKCA, BMP2, CTBP1, PLD1, TCF7, COL4A1, CTBP2, EPAS1, IL8, PIK3CD, STAT1, APPL1, STK4, FZD4, PRKCB, RAD51, MAPK1, SMO, CCDC6, CDKN1B, HIF1A, ETS1, GSK3B, JUN, MAPK9, PTCH1, LAMC1, ABL1, CRK
ErbB signalling pathway	16	1.1412	0.001324	PRKCA, NRG4, ERBB3, PIK3CD, PRKCB, MAPK1, PTK2, CDKN1B, JUN, GSK3B, GAB1, PIK3CA, MAPK9, ABL1, CRK, ABL2
Insulin signalling pathway	21	1.4979	0.001621	SOCS3, PRKAG2, PHKA1, PIK3CD, HK2, PRKCI, PRKAB1, RHOQ, PPP1CB, PPARGC1A, IRS1, PCK1, G6PC2, MAPK1, GSK3B, PIK3CA, MAPK9, PRKAA2, PTPN1, CRK, RAPGEF1
Adipocytokine signalling pathway	13	0.9272	0.00284	PPARA, SOCS3, PRKAG2, PRKAB1, IRS1, PPARGC1A, G6PC2, PCK1, ACSL1, CD36, MAPK9, PRKAA2, ACSL6
Focal adhesion	25	1.7832	0.010295	CAV2, CAV1, TNC, LAMB4, PTK2, PPP1R12A, PIK3CA, TNN, PDGFD, THBS2, RAPGEF1, COL4A4, PRKCA, COL4A1, PIK3CD, PPP1CB, FLNB, PRKCB, MAPK1, GSK3B, JUN, MAPK9, RAP1A, LAMC1, CRK
Renal cell carcinoma	12	0.8559	0.01145	MAPK1, HIF1A, EPAS1, ETS1, JUN, PIK3CD, GAB1, RAP1A, PIK3CA, CRK, RAPGEF1, TGFB2
Aldosterone‐regulated sodium reabsorption	8	0.5706	0.026626	PRKCA, MAPK1, PIK3CD, ATP1B4, PIK3CA, NEDD4L, IRS1, PRKCB
Neurotrophin signalling pathway	16	1.1412	0.034362	IRAK2, PIK3CD, IRS1, RPS6KA6, MAPK1, PSEN1, MAP3K1, JUN, GSK3B, GAB1, PIK3CA, RAP1A, MAPK9, ABL1, CRK, RAPGEF1
Fc gamma R‐mediated phagocytosis	13	0.9272	0.041454	PRKCA, PLD2, DNM3, PLD1, WASF1, PIK3CD, ARF6, ARPC5, PRKCB, MAPK1, PIK3CA, PPAP2B, CRK
TGF‐beta signalling pathway	12	0.8559	0.04935	ACVR2A, MAPK1, ACVR2B, BMP2, SMAD9, ID4, SMURF2, SMAD1, BMPR1B, THBS2, CUL1, TGFB2

**Table 4:** Pathways enrichment and related genes of hsa‐miR‐2117, hsa‐miR‐4494, hsa‐miR‐4502 (three up‐regulated miRNAs)

KEGG_PATHWAY	Count	%	*P*‐value	Genes
Apoptosis	9	1.111111	0.029075	BID, IRAK3, CASP7, IL1RAP, CHP2, PPP3CC, ENDOD1, PPP3CA, BIRC3
Axon guidance	11	1.358025	0.044223	SEMA5A, PAK7, NCK2, PAK2, CHP2, PPP3CC, SEMA3A, PPP3CA, UNC5C, SRGAP1, RASA1

**Figure 2:**
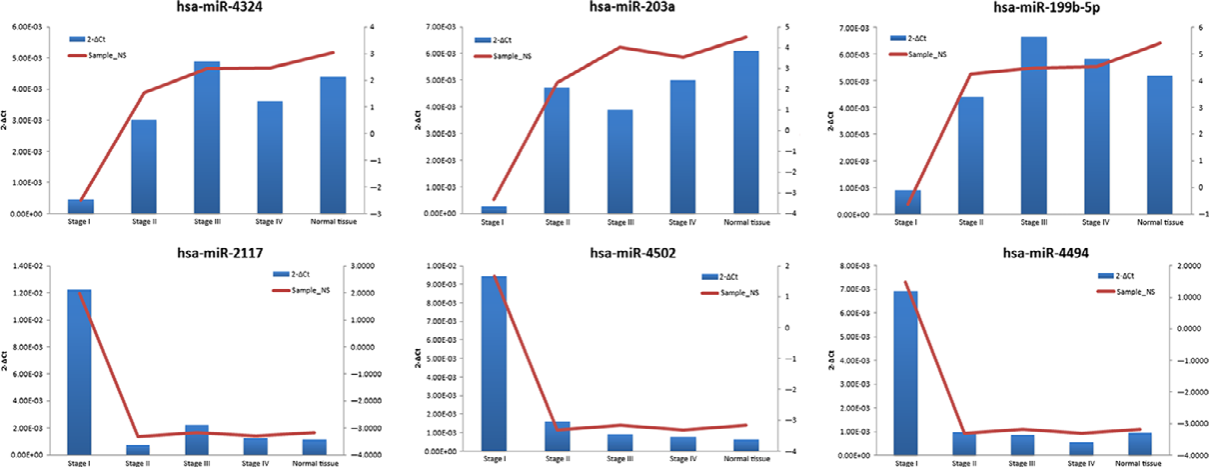
Expression difference of miR‐203a, miR‐199b‐5p, miR‐2117, miR‐4494, miR‐4502 and miR‐4324 in stage I, II, III, IV of NPC and normal nasopharyngeal tissues. The sample‐signal values of miR‐203a, miR‐199b‐5p, miR‐2117, miR‐4494, miR‐4502 and miR‐4324 were detected in microarray and validated by quantitative real‐time RT‐PCR analysis. *Y*‐axis on the left: 2‐ΔCtin RT‐PCR. *Y*‐axis on the right: sample‐signal value in microarray.

**Figure 3:**
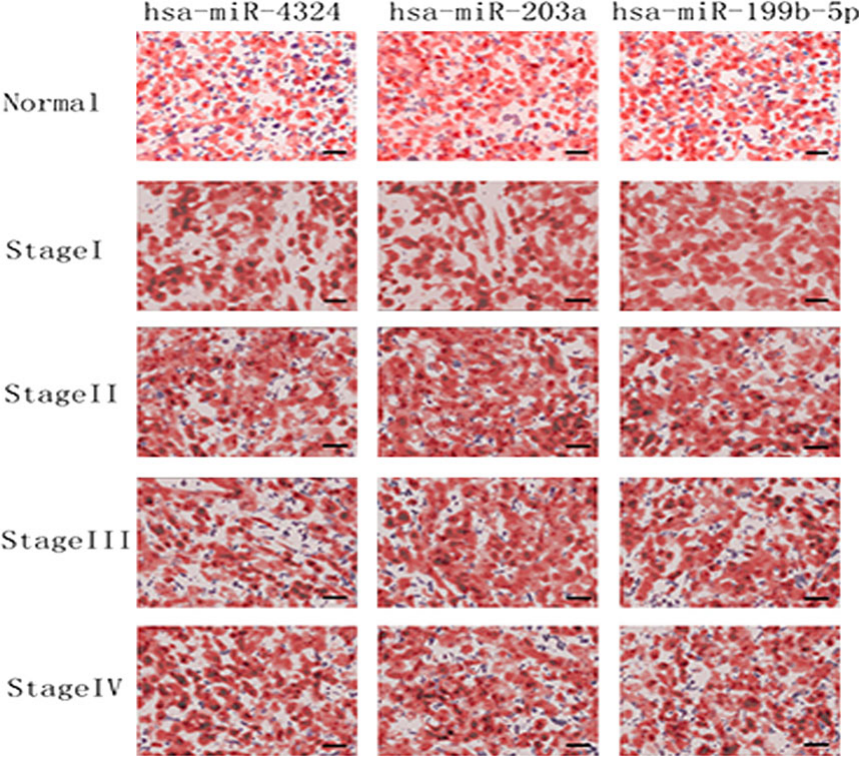
Representative *in situ* hybridization images of hsa‐miR‐4324, hsa‐miR‐203a and hsa‐miR‐199b‐5p. Paraffin sections of NPC and normal tissue were examined using *in situ* hybridization. There were five samples (*n* = 5) for each patient, two patients in one group. The representative images are shown. Hsa‐miR‐4324, hsa‐miR‐203a, hsa‐miR‐199b‐5p probes were used to localize hsa‐miR‐4324, hsa‐miR‐203a, hsa‐miR‐199b‐5p expression at normal tissue, stage I, stage II, stage III, stage IV. MiRNA labelling is blue/purple, and the nuclear counterstain is red. At stage I, all of three miRNAs were expressed at lower level than other groups. There is no significant difference between normal tissue, stage II, III and IV, scale bar = 20 μm.

Pathways in cancer, ErbB signalling, insulin signalling, adipocytokine signalling pathway, focal adhesion, renal cell carcinoma, aldosterone‐regulated sodium reabsorption, neurotrophin signalling, Fc gamma R‐mediated phagocytosis and transforming growth factor (TGF)‐beta signalling were co‐regulated by down‐regulated miRNAs (hsa‐miR‐4324, hsa‐miR‐203a, hsa‐miR‐199b‐5p) (Table 3). Although, three up‐regulated miRs (hsa‐miR‐2117, hsa‐miR‐4494, hsa‐miR‐4502) modulated NPC genesis by axon guidance and apoptosis (Table 4). As the results of pathway enrichment analysis, three down‐regulated miRs were involved in malignant tumour pathways. To verify the reliability of miR array result, quantitative RT‐PCR was carried out to investigate the expressions of hsa‐miR‐4324, hsa‐miR‐203a, hsa‐miR‐199b‐5p, hsa‐miR‐2117, hsa‐miR‐4494 and hsa‐miR‐4502 in stage I, II, III, IV NPC tissues or in normal nasopharyngeal tissues. Consistent with the array data, hsa‐miR‐2117, hsa‐miR‐4494 and hsa‐miR‐4502 were significantly up‐regulated in stage I NPC; whereas hsa‐miR‐4324, hsa‐miR‐203a and hsa‐miR‐199b‐5p were less expressed in stage I NPC (Fig. 2). For further investigation, ISH were performed on another 20 samples at various NPC stages (different from the samples used in microarray). *In situ* hybridization results confirmed low expressions of hsa‐miR‐4324, hsa‐miR‐203a and hsa‐miR‐199b‐5p in stage I NPC (Fig. 3).

### Hierarchical clustering analysis of dysregulated miRNA expression in all NPC stages

**Figure 4:**
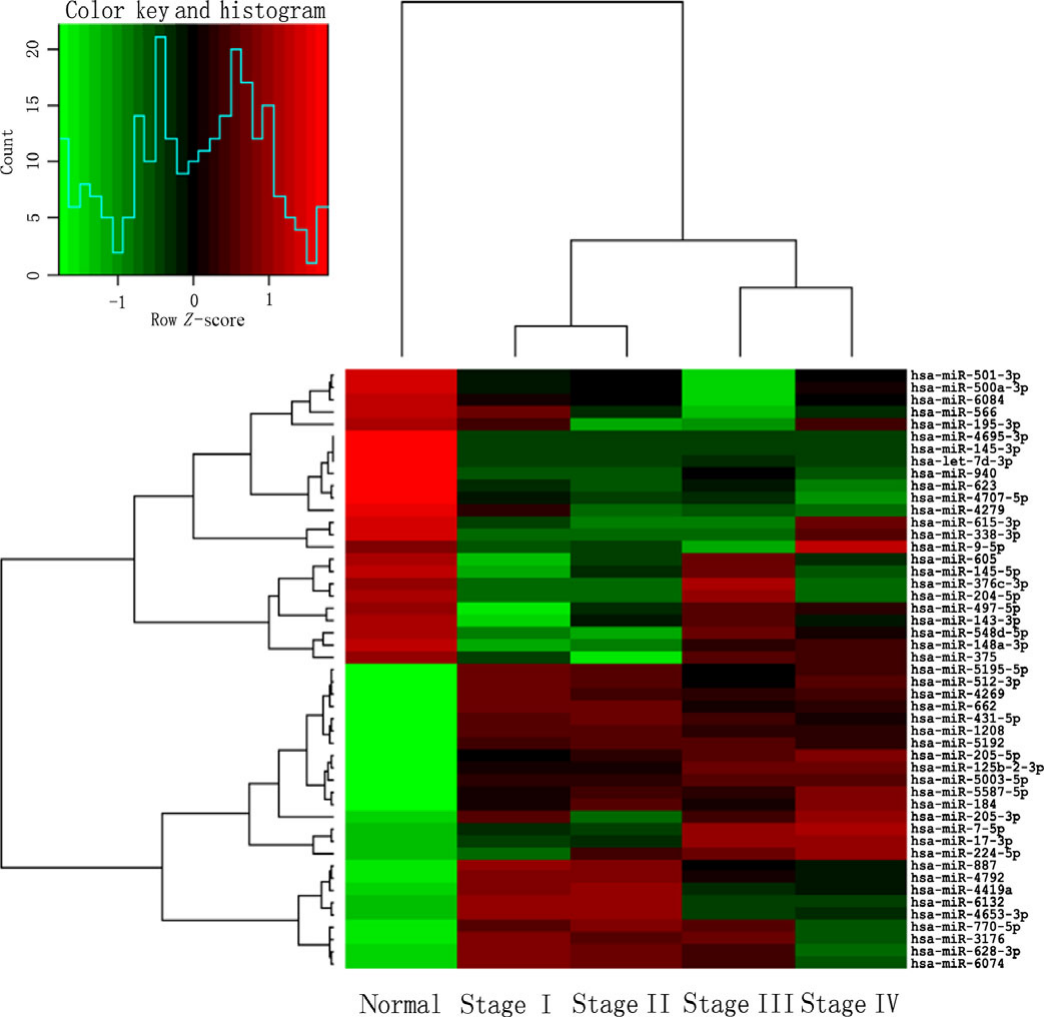
Hierarchical clustering of 49 miRNAs expressed differentially between stages I, II, III, IV of NPC.

Hierarchical clustering analysis of the 49 miRNAs dysregulated in all stages of NPC (The “O” category in Fig. 1) was performed with R software. By Hierarchical clustering analysis, expression diversity of those 49 miRNAs was observed in NPC and normal pharyngeal tissues with visual representation. As shown in Figure 4, comparing with nasopharyngitis tissue, tumour tissues were classified into two groups: stage I and II NPC in one group, and stage III and IV NPC in the other group (*x*‐axis).

### Let‐7 family expression in the microarray data

In the microarray analysis, nine members of let‐7 family were dysregulated according the FC criteria. Five members of let‐7 family (hsa‐let‐7d‐5p, hsa‐let‐7e‐5p, hsa‐let‐7f‐5p, hsa‐let‐7g‐5p, hsa‐let‐7i‐5p) were down‐regulated only in stage I NPC. Three members of let‐7 family (hsa‐let‐7a‐5p, hsa‐let‐7b‐5p, hsa‐let‐7c) were down‐regulated in stage I and II NPC. One member of let‐7 family (hsa‐let‐7d‐3p) was down‐regulated in all stages of NPC (Table 5). The let‐7 family is one of the extensively studied groups of miRs. A previous study revealed that let‐7 (‐a, ‐b, ‐d, ‐e, ‐g and ‐i) were down‐regulated in NPC cells. This resulted in inhibition of cell proliferation through down‐regulation of c‐Myc expression 33. Interestingly, in stage III and IV of NPC, only hsa‐let‐7d‐3p was down‐regulated. Other members of let‐7 family displayed lower expression level in early stages of NPC only (stage I and II).

**Table 5:** The sample‐signal value of let‐7 family in different stages of NPC detected by microarray

Systematic name	Stage I_NS	Stage II_NS	Stage III_NS	Stage IV_NS	Nasopharyngitis_NS
hsa‐let‐7a‐5p	8.102136	9.300925			10.331304
hsa‐let‐7b‐5p	7.5444303	8.989673			10.410756
hsa‐let‐7c	6.1174273	7.374718			8.511084
hsa‐let‐7d‐3p	−3.321953	−3.308076	−3.1660423	−3.301246	0.945101
hsa‐let‐7d‐5p	5.027936				6.8193464
hsa‐let‐7e‐5p	3.8237662				6.2083373
hsa‐let‐7f‐5p	7.753345				9.0592785
hsa‐let‐7g‐5p	7.285065				8.574723
hsa‐let‐7i‐5p	7.248119				8.342013

Sample‐signal values of let‐7 family screened by FC ≥2 and FC ≤0.5 were listed in this table only.

## Discussion

MicroRNAs can be dysregulated in cancer, in which they function as a group to mark differentiation states or individually as bona fide oncogenes or tumour suppressors 34. We selected formalin‐fixed, paraffin‐embedded (FFPE) tissues for analysis because: (*i*) many studies have demonstrated that miRs are minimally affected by FFPE treatment 35, 36, 37; (*ii*) FFPE NPC tissues have been effectively used for diagnosis with haematoxylin and eosin and immunostaining as these samples can be easily collected from clinical tissue banks. Stage I NPC samples (T1N0M0) are rare since enlargement of the lymph nodes occurs as the primary symptom in more than 50% NPC patients 38.

Multiple studies have been performed for miR expression profile in NPC, but specific expressions in different stages of NPC remain unrevealed 24, 25. In our microarray platform, 2006 known miRs were detected. As shown in Figure 1, 449 miRNAs were expressed in NPC at various stages. In 84 miRNAs only dysregulated in stage I NPC, three most down‐regulated miRs, namely miR‐203, miR‐199b‐5p, miR‐4324,were selected for further analysis. In previous studies, those three miRs were found to be down‐regulated in some forms of cancers. MiR‐203 suppresses cancer cell proliferation through the inhibition of SRC in lung cancer 39. ZNF217 and CASK were proved as other targets of miR‐203 and knockdown of ZNF217 and repressing CASK expression attenuated cell proliferation, invasion and migration in colorectal cancer 40, 41. In addition, MiR‐203 can enhances 5‐FU chemosensitivity *via* the down‐regulation of TYMS in colorectal cancer 42 and drive progression of prostate cancer by suppressing LASP1 43. Moreover, miR‐203 is regulated by C/EBPβ‐LIP, E2F, Jun N‐terminal protein kinase and NF‐κB in cancer 18, 44, 45. The latter implies that EBV promotes malignancy by down‐regulating cellular miR‐203 in NPC 18. MiR‐199b‐5p was deemed to be a regulator of the Notch pathway and Sonic hedgehog (SHH) pathway through its targeting of the transcription factor Hairy and enhancer of split 1 (HES1) 46, involved in transcription and post‐transcription regulation in erythroid differentiation 47. In the highly aggressive osteosarcoma cell lines and in the follicular thyroid carcinoma, miR‐199b‐5p was down‐regulated 48, 49. Stable nucleic acid lipid particles that encapsulate miR‐199b‐5p has been used as a tool to impairment of cell proliferation with no signs of apoptosis, which will be the basis for future preclinical studies 50. Hsa‐miR‐4324 was down‐regulated in cutaneous malignant melanoma 51. Employing the DAVID tools, the targets of down‐regulated miRNAs (hsa‐miR‐203a, hsa‐miR‐199b‐5p, and hsa‐miR‐4324) were examined. Pathways in cancer, ErbB signalling pathway, insulin signalling pathway, adipocytokine signalling pathway, focal adhesion, renal cell carcinoma, aldosterone‐regulated sodium reabsorption, neurotrophin signalling pathway, Fc gamma R‐mediated phagocytosis and TGF‐beta signalling were co‐regulated by hsa‐miR‐4324, hsa‐miR‐203a, hsa‐miR‐199b‐5p in stage I of NPC (Table 3). All these pathways were proved involved in tumour formation and progression 52, 53, 54, 55, 56, 57, 58. Those three down‐regulated miRNAs may promote the formation of NPC through these pathways. To avoid variety of pooled samples in microarray, we evaluated the expression of miR‐203, miR‐199b‐5p and miR‐4324 by ISH on FFPE sections from another group of NPC patients. The results of ISH also showed down‐expression of those three miRs. Although previous researches revealer the cancer relevance of miR‐203, miR‐199b‐5p and miR‐4324, we are the first to report those three miRNAs were specifically down‐regulated in stage I NPC. Future studies will focus on the mechanisms underlying how miR‐203, miR‐199b‐5p and miR‐4324 regulates NPC formation.

On the other hand, we analysed three up‐regulated miRs (hsa‐miR‐2117, hsa‐miR‐4502, hsa‐miR‐4494) in stage I NPC. MiR‐2117 has been suggested as a potential bona fide miR in ovarian cancer 59. The predictive targets of miR‐2117 were involved in two important pathways, apoptosis and axon guidance (Table 4). Axon guidance (*i.e*. axon path finding) is a process by which neurons send out axons to reach the correct targets. SEMA3F, an important molecule in axon guidance, is involved in cell adhesion, migration, invasion, and proliferation and inhibits the growth and metastasis in cancer 60, 61, 62. The up‐regulated miRs were also found to suppress apoptosis pathways. An impaired apoptosis often results in formation of tumours 63.

It has been reported that the Let‐7 family associated with the growth and invasion of malignant tumours including NPC 17, 64, 65. Interestingly, we found eight Let‐7 members (namely hsa‐let‐7a‐5p, hsa‐let‐7b‐5p, hsa‐let‐7c, hsa‐let‐7d‐5p, hsa‐let‐7e‐5p, hsa‐let‐7f‐5p, hsa‐let‐7g‐5p, hsa‐let‐7i‐5p) were down‐regulated in early stage of NPC and 1 member (hsa‐let‐7d‐3p) was down‐regulated in all stages of NPC. We speculated that since most Let‐7 family members were dysregulated in early stage of NPC they may be involved in the early formation of NPC.

In summary, we have identified stage‐specific miRs in NPC patients. In this study, miRs specifically dysregulated in stage I NPC. Several biological pathways were identified to be associated with the identified miRNAs. *In situ* hybridization further confirmed the low expressions of miR‐203, miR‐199b‐5p and miR‐4324 in stage I NPC. Although it has been reported that miRs play an important role in NPC carcinogenesis 19, 20, 21, our research advanced the field by identifying stage‐specific miRs. Those miRs are important regulators of NPC formation and can serve as potential therapeutic targets or as biomarkers for early diagnosis. We also found 49 miRNAs dysregulated in every stage of NPC as compared to normal nasopharyngeal tissues. It is likely that those miRs overarch the whole progression of NPC, not just formation.

## Conflict of interest

None.
